# Ten quick tips for bioinformatics analyses using an Apache Spark distributed computing environment

**DOI:** 10.1371/journal.pcbi.1011272

**Published:** 2023-07-20

**Authors:** Davide Chicco, Umberto Ferraro Petrillo, Giuseppe Cattaneo

**Affiliations:** 1 Institute of Health Policy Management and Evaluation, University of Toronto, Toronto, Ontario, Canada; 2 Dipartimento di Scienze Statistiche, Sapienza Università di Roma, Rome, Italy; 3 Dipartimento di Informatica, Università di Salerno, Fisciano (Salerno), Italy; McGill University, CANADA

## Abstract

Some scientific studies involve huge amounts of bioinformatics data that cannot be analyzed on personal computers usually employed by researchers for day-to-day activities but rather necessitate effective computational infrastructures that can work in a distributed way. For this purpose, distributed computing systems have become useful tools to analyze large amounts of bioinformatics data and to generate relevant results on virtual environments, where software can be executed for hours or even days without affecting the personal computer or laptop of a researcher. Even if distributed computing resources have become pivotal in multiple bioinformatics laboratories, often researchers and students use them in the wrong ways, making mistakes that can cause the distributed computers to underperform or that can even generate wrong outcomes. In this context, we present here ten quick tips for the usage of Apache Spark distributed computing systems for bioinformatics analyses: ten simple guidelines that, if taken into account, can help users avoid common mistakes and can help them run their bioinformatics analyses smoothly. Even if we designed our recommendations for beginners and students, they should be followed by experts too. We think our quick tips can help anyone make use of Apache Spark distributed computing systems more efficiently and ultimately help generate better, more reliable scientific results.

## Introduction

Distributed computing and high-performance computing (HPC) systems have become popular in many bioinformatics research groups worldwide, both in academic scientific environments and in companies. Large bioinformatics data, in fact, often can represent and encode a particular biological problem that can be investigated by a principal investigator and their team. By bioinformatics data, we refer to biological and omics data processed through a computer, in contrast to the raw biological data collected in a wet lab. When data are so large they cannot be processed on a singular personal computer, the setup and usage of a distributed computing system becomes necessary to generate meaningful results. For example, distributed computing can be used to align reads of human reference genome in epigenetics studies [1], which is a task that would be difficult or impossible to perform on a personal computer.

To this end, team leaders of bioinformatics laboratories around the world every year decide to allocate resources and funds to create distributed computing environments that can be exploited by bioinformaticians, students, analysts, and collaborators. Even if a distributed computing resource can be useful, often its users did not receive any formal training on how to use it and, therefore, might make mistakes and create problems to themselves and/or to the other users.

In this study, we propose some easy guidelines on how to setup and use an Apache Spark distributed computing system efficiently, by avoiding common mistakes and pitfalls that we noticed or experienced several times in our career.

### Distributed computing, parallel computing, and high-performance computing

Although parallel and distributed computing both fall into the broader realm of HPC, that is, the ability to pool huge computational capability to solve hard problems in a reasonable amount of time, they have significant differences.

Distributed computing is based on distributed systems. By this term, we refer to a collection of independent computer systems connected by a network, modeled as a single supercomputer system thanks to the use of common middleware software. Distributed computing can be used to solve complex problems by breaking them down into smaller problems that are then addressed independently by the nodes of a distributed system. In contrast, parallel computing takes advantage of parallel systems. These systems also exploit huge computational power, but this is achieved by concentrating a large number of computational units on a single machine. In principle, parallel systems would be more efficient than distributed systems for a number of reasons. For example, the different computational units share the same memory space, and communication between them is almost instantaneous. However, distributed systems are far more elastic. A hardware failure that targets a parallel system makes it unavailable, while the same failure that occurs on a node of a distributed system has little effect on its availability.

Another example is scalability. It is possible to increase or decrease the computational capacity of a distributed system simply by increasing or decreasing the number of computational systems used. This is not the case with parallel systems.

Designing, building, configuring, and maintaining a distributed system from scratch can be a challenging task that requires many nontrivial skills and also comes at a significant cost. This may discourage adoption of this technology when one has to carry out analysis like those required in bioinformatics. However, there is a much more convenient shortcut. Instead of building a distributed system, the interested user can simply rent one from cloud computing providers, just for the time needed to perform the proposed experiments. In such a case, no special technical skills are required, and the requested distributed system is ready for operation in a few clicks and in a few minutes.

### The advantages of distributed computing

A distributed cluster is the best choice if one is looking for a computational platform able to scale out when the size of input data increases. If time spent for reading the input dataset is too long, the input/output (I/O) subsystem of the server (multicore shared memory) becomes the bottleneck. A distributed platform decouples the storage from the computing resources leaving each node to process locally stored data without affecting any other node. On the other hand, such a distributed platform requires complex components like Resource Manager (RM), distributed file system, job scheduler, etc. These components are crucial for the cluster resource management and for the correct application execution. They are difficult to implement They are difficult to implement and install for beginners, but there are many commercial proposals that provide computing resources with these components already installed and configured, such as Amazon EMR (Elastic MapReduce), Google Dataproc, Databricks Lakehouse Platform, Cloudera, and many others. These cloud enablers deliver fully managed and highly scalable services for running Apache Hadoop and Apache Spark on cloud resources reducing the installation and the setup effort. We are not inviting to use these platforms and of Dockers, Containers, Singularity, and Kubernetes technologies in this article because we designed these quick tips for beginners, who do not have the advanced skills necessary to correctly configure and use these tools.

We recommend choosing a particular platform only if all its components are clearly described. Do not write the software code to implement them by yourself, since it may result a burdensome, difficult job.

### The context

A study by Giuseppe Agapito [2] described the main applications of distributed computing in computational proteomics, while an article by Terry Disz and colleagues [3] and an article by Shih-Nung Chen and colleagues [4] reported the main challenges of distributed computing usage in computational biology.

In the *PLOS Computational Biology* education series, Jamie J. Alnasir [5] proposed some practical quick tips for an easy usage HPC clusters, and Cole and Moore [6] introduced some quick tips for designing biomedical workflows on cloud computing resources. Although interesting, these tips focus only on Linux-based HPC and on cloud computing workflows, respectively, while our study here refers specifically to distributed computing, giving broader recommendations on how to arrange and utilize a distributed computing resource.

Our guidelines for managing an Apache Spark distributed computing resources, if taken into practice, can help you avoid many headaches and make your computational life easier. Although we wrote our quick tips for beginners and students, we believe they should be kept in mind by experts, too.

## Tip 1: Use the distributed computing resources for your bioinformatics analyses only if it is necessary

Even if the institute or the lab where you work or study provides a distributed computing system, it does not mean that you need to use it for all the bioinformatics analyses that you need to carry out. On the contrary, as simple as it might seem, we recommend you utilize the distributed computing resources only if it is necessary. For example, let us suppose you need to analyze the GSE116660 dataset of microarray gene expression of patients with neuroblastoma [7,8], contained in a tar file of 80 megabytes (MB). You can perform your bioinformatics analysis on your personal computer, if its computational power and its available memory is sufficient, without using the distributed system. On the other hand, there are cases where one has to analyze datasets so big, that they would be intractable on a personal workstation or even a single computing server. Think, for example, of the huge matrices returned by single-cell RNA-seq experiments. These can easily reach hundreds of gigabytes in size, and their analysis, even if also for basic tasks like normalization [9], becomes likely out of reach for many non-distributed computing systems.

This practice, if applied constantly, would leave the distributed system nodes uncongested and more available to all the users and would give you the opportunity to keep your data and scripts at hand on your computer. Generally speaking, when one has a huge amount of genomic data (in terms of gigabytes) in a variety of formats that can be split and processed in smaller autonomous subsets, then distributed computing can be the right technology to use. On the other hand, keep in mind that a software program that works well on your personal computer might not necessarily function on a distributed system, and vice versa.

## Tip 2: If you have the possibility to build a new distributed computing system from scratch, choose Apache Spark

We envisioned the tips of this article for users of distributed computing systems who are interested in doing bioinformatics analyses, but this tip is oriented to researchers who might have the chance to design their own computational environment from scratch by choosing a targeted distributed platform, such as principal investigators or team leaders starting their own labs. For those having this privilege, we recommend Apache Spark [10,11]. In particular, we suggest to pick Apache Spark, with the addition of Apache Hadoop because of its resource scheduling capabilities as implemented by the YARN module.

Apache Spark is an open-source unified analytics engine employed in many applications regarding computational biology [12] and machine learning [13]. Even if no common consensus has been reached in the hi-tech community regarding the best framework for distributed computing, we believe that, without any specific requirements, Apache Spark has some key advantages that make it a better choice than other platforms, such as Apache Hadoop alone and Apache Flink [14–21].

Apache Spark, in fact, results being usually faster than Apache Hadoop alone [22] when the application and the input size exploit the ability to apply the transformations directly in-memory. On the other hand, Apache Flink is stream oriented and this feature makes this tool often more complex to apply to genomic studies based on sequences analysis. Apache Spark has the advantage of providing several abstract data types with the related highly specialized application programming interfaces (APIs) (such as resilient distributed dataset (RDD) [23], dataset, and dataframe) and multiple software packages such as the MLlib library for effective iterative in-memory machine learning computations [24]. Moreover, Apache Spark has a high-level graphical user interface (GUI) to profile the application and allows interactive shell mode. Programs can be written in Scala, R, Python, Java, and Apache Spark SQL [25].

Even if we recommend not using Apache Hadoop as distributed computing framework, we suggest taking advantage of it as cluster manager, since it provides a full set of cluster services like the Resource Manager, a coarse grain scheduler (YARN), a distributed file system, a node failure recovery mechanism, low-level communication monitoring, and a user web GUI. Our general tip, in fact, is about using the Apache Spark framework with Apache Hadoop as cluster manager.

## Tip 3: Use only the framework-native programming language if sufficient, or open source programming languages and software libraries otherwise

### Framework-native programming language

When starting a new bioinformatics project on Apache Spark, you face the decision about which programming language and software to use: In this scenario, this choice may heavily affect the performance and the development time and is not simply a matter of taste. Often, it is driven by the number of necessary functions already implemented by the libraries brought by the language or by our experience gained in different previous contexts.

As Apache Spark has been developed using Scala [26], we suggest you to develop your software code in this framework-native programming language, if its software libraries offer good coverage of your needs.

We define foreign programming languages as all programming languages that were not used to implement the specific platform. Many foreign languages, such as R and Python, can be used to implement Apache Spark tasks, but using Scala allows users to access all the APIs offered by the Apache Spark environment included the public methods of its internal data structures. On the other hand, Python or R can only allow users to access the end user Apache Spark APIs that have been wrapped and exposed by Python or R high-level functions preventing the extension of the features provided by Apache Spark.

**Fig 1:**
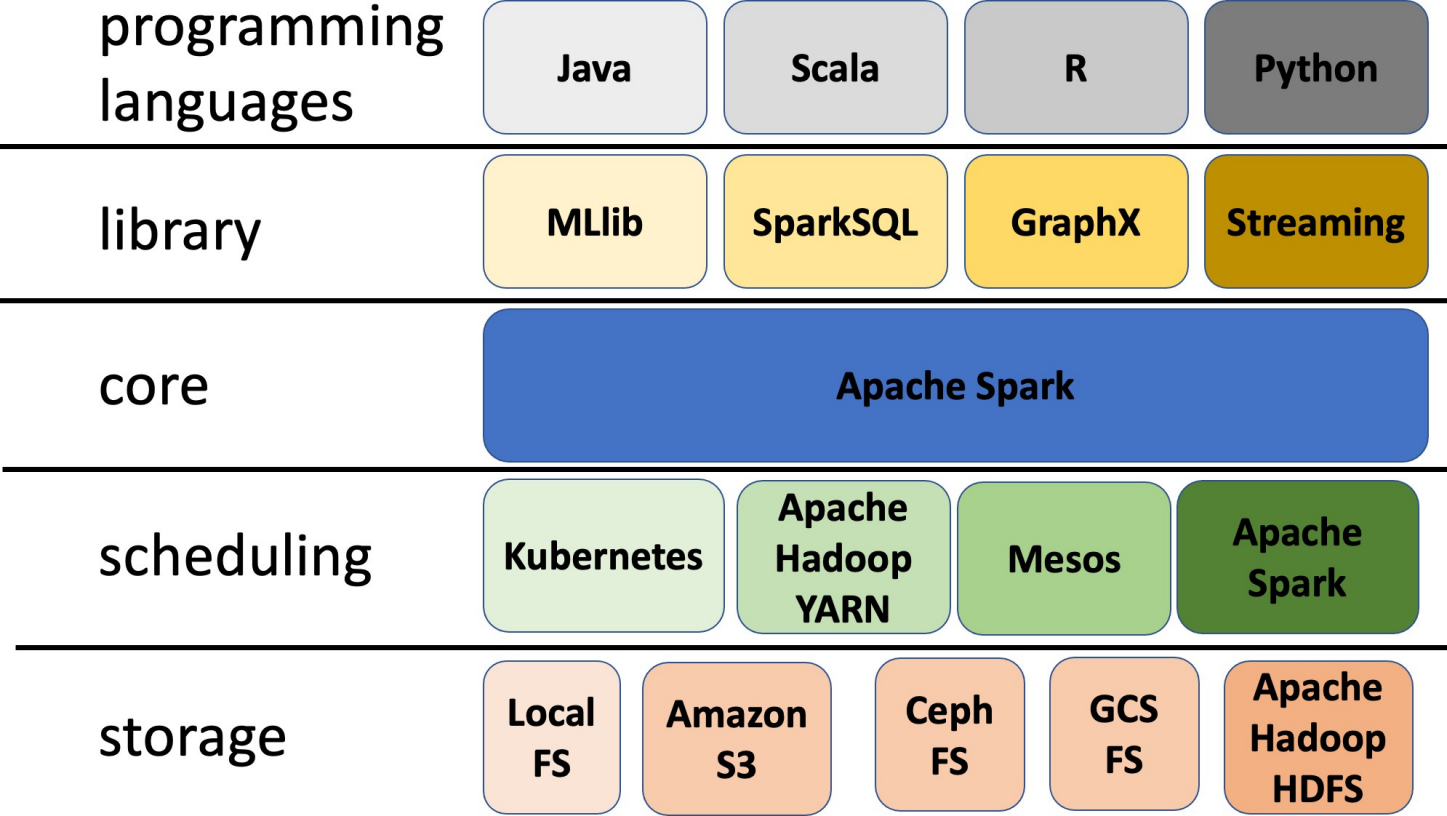
The Apache Spark layered architecture. The colors are used only to distinguish the elements. From bottom to top, the first layer shows some of the most common storage options used by Apache Spark applications to store and retrieve external data: the local file system, the Apache Hadoop HDFS file system, the S3 file system, the Ceph file system, and the GCS file system. The second layer shows the scheduling engines that support the ability to run Apache Spark computations across the nodes of a distributed system: Apache Hadoop YARN, Mesos, Kubernetes, and the cluster manager integrated with Apache Spark. The Kubernetes option has been included despite missing some relevant features, such as resource management and job queues, because it is frequently used in the real world. The third layer shows the core of the Apache Spark framework. The fourth layer shows the standard libraries that are integrated with Apache Spark: SparkSQL, useful for querying very large datasets using a dialect of the SQL language; MLlib, a library of ready-to-use machine learning algorithms and methods; GraphX, a library for representing and processing very large graphs using a distributed approach; and Spark Streaming, a library for distributed processing of streaming data. The top layer lists the programming languages that can be used to write Apache Spark applications.

Choosing a foreign language has another side effect: In fact, the wrappers from developer language to framework-native language may come to the execution of hidden huge operations necessary to translate the basic data types (for example, the way data is stored in memory by the Python or R interpreter) to the one used by the Apache Spark platform or Scala. In other words, even if Apache has developed a language-independent columnar memory format for flat or hierarchical data, called Arrow, the amount of work spent to collect a huge amount of records to the Spark driver (as defined in S1 Fig) from the output of different tasks increase the execution time because data should be translated in a foreign format. We represent the Apache Spark’s stack layers in Fig 1.

Using the framework-native programming language will bring you several additional advantages:

1. A lower overhead in terms of number of translation of data to be processed;
2. A better integration with the platform that will provide you with a lot of framework-native tools to support monitoring and debugging;
3. A better integration with programming tools necessary for the distributed execution;
4. The availability of library highly optimized for the execution on the target platform (such as serializers like Apache Avro [27], Apache Parquet [28], or Apache Thrift [29], which can heavily affect the overall application performance when the amount of data increases).

The third point above is particularly meaningful when the platform hides the distributed computing details. For instance, when your software code is packaged in a Java archive (JAR file) with all its external dependencies, it requires only that the Java virtual machine (JVM) must be installed on each worker node to be executed. On the other hand, Python or R programming environment do not provide a utility like Apache Maven to produce such a self-contained archive. In such a case, the user must take care of installing all the required modules (choosing the right version) on each node of the cluster (Fig 1) and upgrading all the nodes when a new version of a module is required. One possible solution to this problem is to use containers to pack everything needed to run an Apache Spark worker into a single image file, including the software to be run with all its external dependencies. This possibility is recognized by the standard Apache Spark distribution, as evidenced by the availability of a reference Docker image for new installations.

Even though we recommend to develop your software code in Scala on Apache Spark, we know that this programming language is often uncommon in bioinformatics labs worldwide, and, therefore, it might be difficult for someone to learn it from scratch or to find someone who can use it in a bioinformatics team. If learning Scala was too time-consuming for you and nobody in your team was able to use it for software implementation, we recommend settling for Java, since Java software code can be executed on JVMs.

### Open source programming languages

If the software packages of the framework-native programming language within a distributed system are insufficient for your bioinformatics analyses, we suggest you to always employ open source programming languages such as R and Python and to avoid proprietary software. Using open source software would make it easier for you to share code scripts and programs among your collaborators, within your institute and outside of it, and to publish it online, without worrying about license constraints.

R is currently the most used programming language in bioinformatics, especially thanks to the spread of the Bioconductor [30] and Bioconda [31] software suites. Python is currently the most utilized programming language worldwide, including in the machine learning community [32]. Among software programs, it is worth mentioning Galaxy, a popular open source program for bioinformatics analyses [33].

Regarding infrastructure for distributed computing, we suggest the open source project Apache Spark, as explained in the previous tip. Apache Spark provides interfaces to R and Python through SparkR [34] and PySpark [35]. For operating systems, we recommend Linux Ubuntu, both for personal computers and for servers. Caveat emptor: At the beginning of each bioinformatics project, make sure that all the software packages needed for your analyses are installed on each node of the distributed computing system and that they are all aligned to the same package version. For example, if you know that your R script uses the GEOquery software library version 2.64.2 from the Bioconductor project [36], before executing it, make sure that exactly the GEOquery version 2.64.2 is installed on each node of the distributed computing system you use. If this package is absent from one of the nodes or is installed with a previous version, errors will happen during the analysis execution.

## Tip 4: Keep the data files compressed

Genomic data can easily reach huge sizes. However, some popular encoding formats (for example, FASTA and FASTQ) are space inefficient. Here, data compression is a standard solution for storing data on persistent devices while avoiding wasted space. The usual approach to data analysis assumes that input data are uncompressed. Thus, before a compressed dataset is loaded, it must be decompressed. This means that a (typically enormous) compressed file is loaded into memory, where it is decompressed into a (usually huge) file that is progressively saved back on persistent storage. When finished, the decompressed file is loaded into memory again for analysis. Indeed, storage devices tend to have access times and latency that can be orders of magnitude slower than those of internal memory. As a result, repeatedly saving and loading large amounts of data can take more time than the analysis itself, in some cases. A solution is to avoid prior decompression altogether (especially for datasets that only need to be processed once) and, instead, perform this operation directly in memory at analysis time. In a nutshell, when performing a computational analysis on a distributed system, do not decompress your compressed data files in advance.

In distributed computing frameworks like Apache Spark, reading compressed files can be done using input readers that support standard compression formats and can transparently read and decompress compressed datasets (on the fly) [37]. A word of caution should be spent here about nonsplittable compression formats. When they are used, the contents of an archive can be decompressed only if the compressed file as a whole is available on a single computer. This may imply a severe performance overhead, especially when working with very large compressed files. Instead, splittable compression formats, on the other hand, fit well with Apache Spark’s distributed approach, since each worker node can decompress a portion of a compressed archive on its own, without interacting with the nodes that contain the remaining portions.

Some recent advancements in this area [38] provide the possibility to easily extend this capability to support either lossless or lossy compression formats specialized for genomic data, such as DSRC [39] and FQZComp [40], even when these are not natively splittable.

Developing ad hoc input-reading functions is also an option that we suggest you consider (Tip 5). However, in the general case, we recommend preserving the original format of compressed files as their preliminary decompression or recompression using another format may have a very negative impact on the overall analysis time.

## Tip 5: Make your input functions specialized to the formats preserving the original input representation

Always encode your data defining your own specialized data types. Whenever you are designing your application from scratch, consider that it is always convenient to encapsulate in your classes the basic functions in charge to process input data instead of using generic data types (and functions). For example, genomic sequences could be represented by strings, but this representation is inefficient for two reasons: First, each nucleotide will be coded in memory with 8 bits (even 16 in some cases) instead of 2 bits; second, this redundant representation could introduce errors if characters other than those allowed are entered in the genomic sequence.

Moreover, often your application must read a huge input from storage. In this case, it is always worth investing your programming effort to implement specialized input functions (called input reader according to the Apache Hadoop terminology). To manage your data, always use either specialized readers for standard input formats, or otherwise proprietary formats based on extensible binary formats, if necessary. In this study, we recommend using only open source programming languages, software programs, and formats (Tip 3). However, if there are no open source formats to save specific files preserving all the information, it is okay to employ proprietary formats.

**Fig 2:**
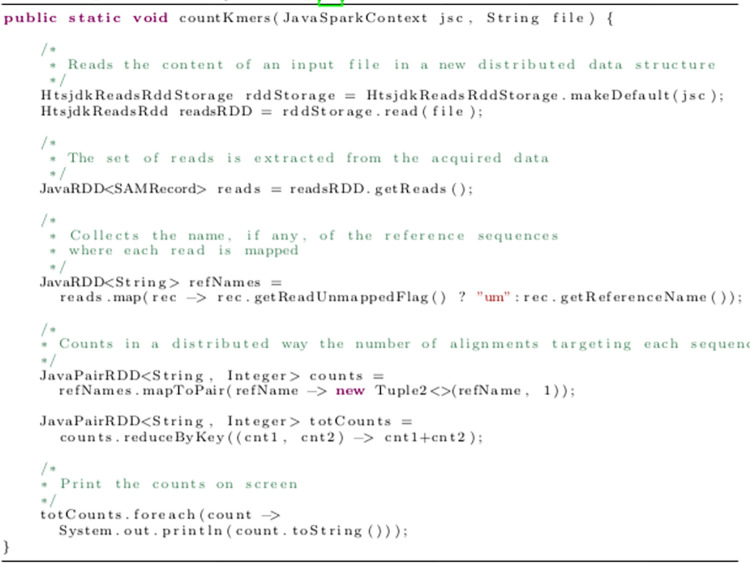
The Java source code of an Apache Spark–based distributed alignments counter implemented using the Disq [44] framework.

So, if you have to read data in standard input formats, you must implement your functions for hiding and encapsulating all the characteristics of your input format. If, on the other hand, you can choose how data are stored on disk, it is necessary to use an extensible binary format such as Apache Avro [27], Apache Parquet [28], or Apache Thrift [29]. In this case, you must consider that each record is a structure stored in a sequence file. When you have to read or write huge amounts of data, the JavaScript Object Notation (JSON) and, even worse, the eXtensible Markup Language (XML) simply cannot be used for this purpose because of the enormous effort (in terms of central processing units cycles) spent for parsing every record [41,42]. Specific libraries to handle genomic files in particular formats are also available on Apache Spark: Hadoop-BAM [43], Disq [44], Glow [45], Hail [46], Sequila [47], and ADAM [48], just to mention a few. We report in Fig 2 the case of a distributed Apache Spark alignments counter built using the Disq [44] framework and able to process BAM/CRAM/SAM files.

For example, considering the k-mer count problem for sequences in FASTA format [49], a specialized input reader could provide filtered input data to the application containing only the representations of the nucleotide sequence without any foreign character such as newline or linefeed and, therefore, without requiring the developer to worry about the short or long format options [50–53].

In this way, the development of software code will be much faster (and error free) while the maintenance for the evolution of the supported formats will be more direct and effective. Implementing your input readers can be a huge opportunity to separate the data processing code from the code that performs I/O partitioning the data on different nodes.

## Tip 6: Partition your data fairly

Computation occurs where data are. Also, the more data you have, the longer their processing time will likely be. In a distributed setting, this roughly translates in the need of ensuring that all the computation units are fed with input data requiring, approximately, the same processing time. In many cases, it is enough to partition an input dataset in n parts of the same size and then send for processing each part to one of the n computing units being used.

However, the processing time of a data batch may depend on several factors other than its size. For instance, when building a distributed algorithm for counting the k-mers in an input genomic sequence, one should consider that some k-mers tend to be much more frequent than other ones [54,55]. So, the computing nodes responsible for those k-mers tend to have much more work to do than other ones. In such a case, an optimal partitioning scheme should know in advance the frequency distribution of the k-mers being counted (or at least an approximation of it), for implementing a well-balanced partitioning scheme. Ideally, one could use a problem-aware strategy able to return a partitioning for an input dataset yielding a uniform workload distribution. This issue has been considered in many contributions [56,57], and a popular solution is to determine an approximation of the k-mers distribution by running the counting algorithm on a small sample of the input data. Then, use the outcoming experimental k-mers distribution to derive a well-balanced partitioning scheme.

In the more general case, it is possible to use the user interface (UI) provided with Apache Spark to analyze the way data is split in blocks and processed at each step of a distributed algorithm and derive useful information about how this partitioning can be improved to achieve an even distribution of workload.

Users of Apache Spark can also consider utilizing Adaptive Query Execution [58], which might help by automatically optimizing the number of partitions and skew joins.

## Tip 7: Keep in mind that more computing units do not imply faster executions

Intuitively, the more computing units one can employ in a distributed computation, the shorter the consequent execution time. This empirical rule tends to be true for embarrassingly parallel problems, where it is easy to decompose a starting problem into smaller problems that can be solved each by each computing unit, independently of the others. But that is not always the case.

In fact, there are several factors that come into play and influence the actual execution of a distributed computation. Some of these factors concern the time required to move data over the network, to reach nodes where it will be processed, as well as the time required to collect the result of this processing. Indeed, scattering small portions of data over a large number of computing nodes may become self-defeating, as the data transmission time might easily overcome the performance gain arising from the distributed execution. This problem can indeed be alleviated by using distributed file systems, like HDFS, to initially scatter the content of a dataset. However, it is still true that, during a Spark execution, there may be still need of moving data from a computing unit to another one, when performing operations requiring scattered data to be aggregated and transformed because of a Reduce operation.

Conversely, keeping the data in a few spots may completely eliminate the need for expensive data transmission operation, even if at the cost of a reduced parallelism. For example, this situation happens when evaluating the pairwise alignment-free distance between the elements of a large genome collection [59]. Here, increasing the number of computing nodes reduces significantly the time required to evaluate distances between genomes but requires as well much longer executions due to the time needed to transmit genomic data from each node to the other ones over the network.

In a few words, we recommend you experiment with the geometry of your distributed system by assessing the trade-off between the usage of a larger number of computing units and the increased communication overhead that this could imply. The most relevant parameters to take into account for this purpose are those related to the number of computing nodes being used, the number of computing units to use on each node, the number of Spark executors running on each of these units, and the number of partitions used to scatter input data over distributed data structures. For a more extensive review of these parameters, we refer the interested reader to [60].

## Tip 8: Properly tune your cluster configuration

Apache Spark is often thought to automatically provide the services to run the application on a cluster. However, after implementing an application, it is always necessary to dynamically tune Apache Spark’s behavior on the specific instances of both the available computing resources (cluster architecture) and the input dataset characteristics (size, structure, etc.), for performance optimization goals. This operation allows one to tailor the available resources to the application context in order to exploit most of the opportunities provided by the programming environment, such as data locality. So, before implementing an Apache Spark program, it is crucial to understand how Apache Spark will map it on its underlying execution model [61,62].

Although all the other tips of this study are meant for beginners, this topic requires a deep knowledge of distributed computing. Beginners can skip this tip and pass it to their systems administrators, while those interested in a complete understanding can find a detailed description of the Apache Spark execution model in S1 Text.

### Apache Spark does not strictly follow the Apache Hadoop model

A common mistake is to extend the Apache Hadoop configuration principles to the Apache Spark framework too. In fact, while Apache Hadoop has been designed to execute one (Map or Reduce) task for each container, in the Apache Spark environment, the Driver automatically tries to launch as many tasks as possible in a single container (according to the available resources) exploiting the multithread capability of the JVM without any extra effort of the application developer.

Therefore, instead of configuring several small containers on each node, it is much more efficient (and comfortable for the user) to have one or a rather limited number of containers on a single node. In this discussion, the following three aspects must be considered: the JVM initialization is extremely slow and its memory footprint is relevant; memory fragmentation may often lead to unused memory holes; and configuring many containers makes the resource balancing much less efficient.

On the other hand, containers with a memory size bigger than 32 gigabytes (GB) can lead to an extra effort during garbage collection operations. So, when your cluster has nodes with a huge amount of memory, configuring multiple containers can help to reduce the overhead.

### Monitoring and profiling your application is the first choice

When the application is ready to run, it is always necessary to profile the entire execution with dedicated tools both internal and external to the Apache Spark environment. On the Apache Spark side, you must consider the monitoring and instrumentation tools provided by the Apache Spark environment itself. During the execution (and also after the execution if the history server has been started), a web UI can be used to grab useful information about the following: the list of scheduler stages and tasks; a summary of RDD sizes and memory usage; specific environment configuration; and information about the running executors.

On the other hand, Apache Hadoop and Apache Spark during the job execution produce a reach set of metrics that can be collected (via REST API or Java Management Extensions JMX interfaces) by specialized tools like Apache Prometheus [63] or SparkMeasure [64].

It can be also useful to check the effective resource usage on each node during each stage of the job execution, with resource-oriented monitoring tools like Zabbix [65] discovering any bottlenecks that result in unused resources or under estimated running time and checking how the application model fits with the available resources.

## Tip 9: Run your bioinformatics analysis on a toy subset of your data first, to test the functioning of your distributed computing system

A common mistake of beginners, when launching a bioinformatics analysis for the first time, is executing it on the whole dataset. This decision might start a software execution that can take hours or days and, in case of execution error or configuration error, could make the bioinformatician waste a lot of time.

A general common practice in computer science to keep in mind is to generate a small toy subset (derived from the complete dataset), to run the bioinformatics analysis that could last few minutes, and, eventually, to check that everything went well and no errors were generated during the usage of the distributing computing resources. Of course, the final results of these executions would have no scientific meaning but would allow you to verify that your software was developed and designed without mistakes. Instead, if errors were generated, handle them and fix their corresponding bugs in your script.

Once you complete the execution of your bioinformatics analysis on this toy subset without mistakes, you could relaunch the software execution on the whole dataset. When generating the toy subset, make sure the data elements are selected randomly, and the execution does not take more than 15 minutes. Randomly pick 0.1%, 1%, or 10% of the whole dataset. This toy subset should be small, of course, but it also should large enough to let you test the computational resources of the distributed system. Make sure your output files contain the suffix “toy,” such as test_results_2022-05-03_h1434_toy.txt, for example.

## Tip 10: Document everything and software profile everything

Documentation is a key pillar of each successful project, not only in bioinformatics and not only in scientific research [66]. To this end, we suggest you to keep a notebook where you document all the aspects of your daily work: which commands, which libraries, which data, which methods you use, and the reasons why you picked them [67]. Write down any dirty trick you need to use to make software work, if applicable. And take note of your scientific decision, too: Why these data? Why that method? What is the scientific question we are investigating here? Document your software, by writing explanations related to the main functions and commands within the code files. Having documentation detailed and complete will be invaluable later, especially when you and your colleagues will write a scientific paper regarding your bioinformatics project [68]. Writing detailed documentation is the best gift you can do to your future self [69–71]. If documentation written by humans is important, so it is the documentation written by computers. This is why the second part of our advice is to software profile everything [72]. Make your software monitor all the resources and save logs of everything happening during its execution: which partitions are used and when, which dataset files are read and when, etc. This software profiling documentation will be useful, especially in case some error happens during the software execution. Software profiling is also crucial to identify possible performance bottlenecks in the execution of a software [73]. These bottlenecks could be caused by a variety of reasons like a misconfiguration in the underlying distributed system, an inefficient algorithmic approach to a problem being considered, or a bad implementation. By profiling its execution, it is possible to pinpoint where a code spends most computational resources, and what it is doing.

## Conclusions

With the exponential growth and availability of bioinformatics data, distributed computing resources have become pivotal in many computational biology research groups worldwide. Even if important, the arrangement and the usage of distributed computing systems might not be easy, especially for biologists and medical principal investigators, who usually lack formal training on these topics.

In this context, we propose these ten guidelines on how to set up and how to use an Apache Spark distributed computing environment and resources to analyze bioinformatics data, by avoiding common mistakes that we experienced or saw in our past projects. We designed our guidelines for beginners, students, biologists, and unexperienced users, but we believe they should be kept in mind by experts, too. We believe our quick tips, if taken into practice correctly, can guarantee a better and more efficient usage of Apache Spark distributed computing clusters, ultimately contributing to generate more robust results and outcomes.

## Supporting information

S1 Text Description of the Apache Spark framework. (PDF)

S1 Fig The Apache Spark computational model. Here, we depict the Apache Spark computational model and how a user job and its related tasks are executed on the underlying cluster managed by Apache Hadoop YARN. When the user launches their Job, first, Spark starts a dedicated JVM to execute the Driver, which manages the so-called Spark Context. Then, it splits the input applying the programmed Spark API (local or wide transformations on input RDD) and planning a list of tasks (orange boxes). Each task is executed by an executor (the green boxes) running on a node of the cluster according to the resource scheduled by the Hadoop Resource Manager. Each executor is executed by a dedicated JVM and may run multiple tasks concurrently. Each cluster node (yellow boxes) may be configured to run several executors (each one in a Hadoop Container). The resources are managed by the Resource Manager, which monitors container status (green arrows), while the task executions and the related I/O are controlled by the Driver (blue arrows). API, application programming interface; I/O, input/output; JVM, Java virtual machine; RDD, resilient distributed dataset.
(PDF)
