# Supplementary material for: Ten quick tips for bioinformatics analyses using an Apache Spark distributed computing environment
Source: PLoS Comput Biol. 2023 Jul 20;19(7):e1011272. doi: 10.1371/journal.pcbi.1011272 (PMC10358940; doi:10.1371/journal.pcbi.1011272)
Supplement: S1 Text — (PDF) [file pcbi.1011272.s001.pdf]

S1.1 The Apache Spark framework

When your application is executed through the command `spark-submit` a single process called Driver starts on one node of the cluster and it is in charge of splitting the user application in smaller execution units called tasks. The cluster manager Apache Hadoop YARN and the Application Master, according to the Apache Hadoop terminology, will schedule the execution of these tasks on the available executors (S1 Figure).
When the Hadoop cluster is configured, the user, depending on the application requirements, should/must define container sizes (JVM heap size for example) and the number of cores for each container. These parameters are particularly meaningful for Hadoop because they determine the parallelism degree for each node. For Spark-based clusters the Driver is able to exploit the JVM multitasking capability running multiple tasks in the same container.
The user can modify the parameters described in this section either permanently changing the Hadoop configuration files or temporarily for the job execution assigning the requested values to the corresponding environment variables. The Driver controls the high-level flow of work that needs to be done. This work is carried out by the executors in the form of tasks. Whereas the requested resources are available, the Driver can ask to an executor to run multiple tasks concurrently throughout its lifetime. When Apache Spark is executed on the top of Apache Hadoop each executor runs in a dedicated container created and managed by the Hadoop RM.

A concrete operating scenario. For instance, considering a cluster with 16 nodes with 32 GB of random-access memory (RAM) and 8 virtual cores (VCores), a good starting configuration is one container per node with 30 GB¸ of RAM and 7 VCores.
This is obtained by assigning the following two properties in the yarn-site.xml Apache Hadoop configuration file:

`yarn.nodemanager.resource.memory-mb` = 30720
`yarn.nodemanager.resource.cpu-vcores` = 7

The first value implies that Apache Hadoop is not requiring all the available memory, leaving a minimal amount to the host operating system and to the other local processes.
Similarly, it is not worth to allocate all the available cores for the Apache Spark task execution, but at least one must be reserved for local processes first of all for the Hadoop distributed file system (hdfs) daemon for the read and write operations on the distributed file system. Otherwise the race condition to acquire the spare resources will slow down all the I/O operations required by the running tasks therefore getting worse overall performance difficult to analyze and to correct.
In such a case the `spark-submit` command parameters will be:

```
--executor-memory = 27.9g
--num-executors = 15
--executor-cores = 7
```

Where:
• `executor-memory` is equal to 97% yarn.nodemanager.resource.memory-mb. In fact, the value of the `spark.yarn.executor.memoryOverhead` property is added to the executor memory to determine the full memory request to Apache Hadoop YARN for each executor. Its default value is max(384, .07 × spark.executor.memory).

• `num-executors` is equal to the product of n (the number of nodes of the cluster) and m (the number of containers per node, 1 in this example), minus 1 which is reserved for the Driver.

• `executor-cores` is the maximum number of tasks run concurrently on each executor and therefore the maximum number of cores exploited by a single container. In this example this implies that on each cluster node one core is not considered by the scheduler (Apache Hadoop YARN) and by the local Hadoop RM.
