# Supplementary material for: Ten quick tips for bioinformatics analyses using an Apache Spark distributed computing environment
Source: PLoS Comput Biol. 2023 Jul 20;19(7):e1011272. doi: 10.1371/journal.pcbi.1011272 (PMC10358940; doi:10.1371/journal.pcbi.1011272)
Supplement: S1 Fig — Here, we depict the Apache Spark computational model and how a user job and its related tasks are executed on the underlying cluster managed by Apache Hadoop YARN. When the user launches their Job, first, Spark starts a dedicated JVM to execute the Driver, which manages the so-called Spark Context. Then, it splits the input applying the programmed Spark API (local or wide transformations on input RDD) and planning a list of tasks (orange boxes). Each task is executed by an executor (the green boxes) running on a node of the cluster according to the resource scheduled by the Hadoop Resource Manager. Each executor is executed by a dedicated JVM and may run multiple tasks concurrently. Each cluster node (yellow boxes) may be configured to run several executors (each one in a Hadoop Container). The resources are managed by the Resource Manager, which monitors container status (green arrows), while the task executions and the related I/O are controlled by the Driver (blue arrows). API, application programming interface; I/O, input/output; JVM, Java virtual machine; RDD, resilient distributed dataset. (PDF) [file pcbi.1011272.s002.pdf]

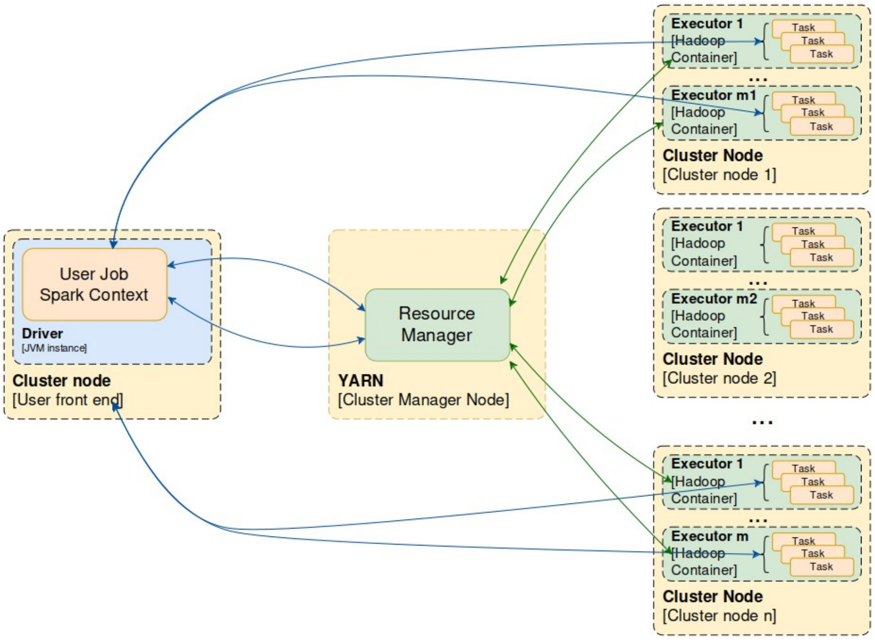

S1 Figure. The Apache Spark computational model. Here we depict the Apache Spark computational model and how a user job and its related tasks are executed on the underlying cluster managed by Apache Hadoop YARN. When the user launches their Job, first Spark starts a dedicated JVM to execute the Driver which manages the so called Spark Context. Then it splits the input applying the programmed Spark API (local or wide transformations on input RDD) and planning a list of tasks (orange boxes). Each task is executed by an executor (the green boxes) running on a node of the cluster according to the resource scheduled by the Hadoop Resource M anager. Each executor is executed by a dedicated JVM and may run multiple tasks concurrently. Each cluster node (yellow boxes) may be configured to run several executors (each one in a Hadoop Container). The resources are managed by the Resource M anager which monitors container status (green arrows) while the task executions and the related I/O are controlled by the Driver (blue arrows).
